# The Association between Birth Weight and Gestational Age and Asthma in 6-7- and 13-14-Year-Old Children

**DOI:** 10.1155/2016/3987460

**Published:** 2016-06-09

**Authors:** Zamani Raheleh, Alikhani Ahmad, Heydarzadeh Abtin, Zare Roghaye, Hashemain Sara, Rahimi Siavash

**Affiliations:** ^1^Department of Pediatrics, Faculty of Medicine, Mazandaran University of Medical Sciences, International Branch, Basij Avenue, P.O. Box 4815733971, Juybar, Sari, Iran; ^2^Infectious Diseases Department and Antimicrobial Resistance Research Center, Mazandaran University of Medical Sciences, Basij Avenue, P.O. Box 4815733971, Juybar, Sari, Iran; ^3^Community Medicine Department, Gilan University of Medical Sciences, Rasht, Iran; ^4^Epidemiology & Biostatistics Department, Tehran University of Medical Sciences, Tehran, Iran; ^5^Ramsar Branch of Mazandaran University of Medical Sciences, Basij Avenue, P.O. Box 4815733971, Juybar, Sari, Iran

## Abstract

*Background*. Previous studies that assessed the role of birth weight and gestational age in the risk of asthma have been conflicting.* Objectives*. To examine the association between birth weight and gestational age and symptoms of asthma.* Patients and Methods*. Subjects were 6656 school children of ages 6-7 and 13-14 years from urban districts of Mazandaran, Iran. ISAAC questionnaires were used.* Results*. There was an increased risk of “wheeze ever” in both age groups with birth weight under 2.5 kg and in all subgroups of low birth weight (LBW). Birth weight more than 3.5 kg was associated with lower risk of “severe asthma” in age group 6-7 years. With respect to gestational age, higher risks of “wheeze ever,” “asthma ever,” and “night cough in the past 12 months” were found in age group 13-14 years born before 37 weeks and the risk of “severe asthma” was higher in younger group (6-7 years). A lower risk of "asthma ever" was also found in 6-7-year-old children and 13-14-year-old girls who were born after 40 weeks.* Conclusions*. This study showed that there is a direct relation between “wheeze ever” and LBW and an inverse relation between risk of “severe asthma” and birth weight more than 3.5 kg.

## 1. Introduction

Asthma is a known global public health issue. Its prevalence in different countries ranges from 0.7 to 18.4% [1]. In Iran, the prevalence of “current asthma” has been reported to be about 12% and the prevalence of clinical asthma is 5%. Asthma is the most prevalent chronic disease of children which affects boys more than girls. The incidence in females begins to rise at puberty. It has been noticed that, by the age of 40, more women than men have asthma. Like other atopic diseases, the prevalence has risen dramatically in recent years and it is still on the rise [1, 2]. The cause of the increasing prevalence of atopic diseases has been sought in numerous studies. A close relationship has been found between the prevalence of diseases and contact with allergens, respiratory infections, cigarette smoking of parents [3–6], and obesity [7, 8], but more research is necessary to verify the role of these agents. Attention has increasingly been focused on the prenatal and perinatal period to identify factors which may help predict the development of asthma and wheezing associated lower respiratory illnesses.

Maternal infections [9–11], pregnancy complications [12], uterine factors, abnormal presentations [13, 14], and mode of delivery [15–21] have been proposed as possible risk factors for asthma in older ages, but the results are inconclusive and still call for more research in this area [22–24].

In some studies, the relationship between birth weight and prematurity has been proposed as a perinatal risk factor for asthma [25–27]. For an instance, in a meta-analysis in the year 2014, 28 to 34% increase in prevalence of asthma was reported in children with low birth weight [28]. The results of the aforementioned studies are not homogenous, such that, in some studies, prevalence of asthma in under 5-year-old children with low birth weight was higher, but other studies did not show such results. Also, in some studies, there was a positive correlation between high birth weight and the prevalence of asthma. Results of many researches are not comparable, because of the difference in asthma definition and classification of weights.

One obstacle to the investigation of population differences had been the lack of a suitable and generally accepted method of measuring the prevalence and severity of asthma in children which could be used worldwide. Another obstacle was the absence of a coordinated research program to obtain and analyze comparative data. The International Study of Asthma and Allergies in Childhood (ISAAC) program was developed in 1991 to evaluate the prevalence and severity of asthma, rhinitis, and eczema in children living in different centers, to describe the data and make it be comparable, and to provide a framework for further etiological researches about factors affecting these diseases.

There is large variations in the prevalence of symptoms of asthma and allergic disease throughout the world (more than 20-fold between centers) and between people of similar genetic origin living in different environments; it was concluded that the environmental factors are the cause of these large variations such that the prevalence of “wheeze in the past 12 months” is 2.1 to 32.2% in age group 13-14 years and 4.1 to 32.1% in age group 6-7 years. The highest percentage belongs to English speaking countries and Latinos [29].

A systematic review in 2014 which is based on the results of ISAAC phase 3, concerning the correlation between birth weight and the prevalence of allergic disease's symptoms in children of 6-7 years of age, showed that low birth weight (<2.5 kg) was associated with an increased risk of symptoms of asthma (current wheeze, odds ratio = 1.20; 95% CI = 1.12–1.30). High birth weight (birth weight ≥ 4.5 kg) was not associated with any of these outcomes [30]. Since studies are not homogenized in terms of asthma symptoms definition and methods of analysis, consistent results about correlation between birth weight and gestational age with prevalence of allergic symptoms are not enough. Also comparison was mostly based on “less than 2.5 kg,” “2.5–4 kg,” and “more than 4 kg.”

This study has evaluated the association between gestational age (preterm, term, and postterm) and birth weight with the prevalence and severity of asthma symptoms with the use of ISAAC questionnaires, which has been done in 6-7- and 13-14-year-old children in primary schools, Mazandaran, Iran.

## 2. Method

Participants were students from urban schools of Ramsar and Tonekabon (West Mazandaran, Iran). They were of two different age groups (6-7 and 13-14 years). All subjects had written consent from their parents. ISAAC written questionnaires for prevalence were filled by parents of younger age group, equivalent questionnaires were filled by children of age group 13-14, and the risk factor questionnaires were filled by parents in both age groups.

Symptoms of asthma and wheezing were evaluated as “wheeze ever,” “wheeze in the past 12 months” (current wheeze), “asthma ever,” “wheeze during or after exercise in the past 12 months,” and “night cough in the past 12 months.”

Diagnosis of severe asthma was based on the presence of any of the following criteria:

- Four or more attacks in the past 12 months.
- Sleep disturbance in one or more nights per week due to difficulty in respiration in the past 12 months.
- Difficulty in pronouncing two-word phrases in the past 12 months.

A sample size of 3000 was chosen, which gives the following power: (1) prevalence of wheezing: if the true one-year prevalence of wheezing is 30% in one center and 25% in another center, with a sample size of 3000, the study power to detect this difference will be 99% at 1% level of significance; (2) severity of wheezing: if the true one-year prevalence of severe asthma is 5% in one center and 3% in another center with a sample size of 3000, the study power to detect this difference will be 90% at 1% level of significance.

Descriptive results were reported using frequency and percent. Odds ratios (ORs) were calculated with generalized linear models, with a binomial distribution and logit link, adjusted for sex to fit for all participants. ORs more than 1 show more risk in contrast to reference group. The symptoms were modeled as function of birth weight/gestational age, and sex and their interaction. If the sex-by-treatment interaction was not statistically significant, the model was refitted with only main effects for treatment. The reference category was 2.5 to 3.5 kg for birth weight and 37 to 40 weeks for gestational age. First models were fitted as a function of birth weight by three groups (<2.5, 2.5–3.5, and >3.5 kg) and to know what happen below 2.5 kg. The models were refitted as function of birth weight by five groups (<1.5, 1.5–2, 2–2.5, 2.5–3.5, and >3.5 kg). The significance level was 0.05. The sample size was at least 3000 for each age group which was chosen based on ISAAC manuals.

## 3. Results

6656 students participated in this study: 3102 belong to age group 6-7-years and 3554 belong to age group 13-14 years. Age group 6-7 years consists of 46.7% boys and 53.3% girls and age group 13-14 years consists of 51.9% boys and 48.1% girls. In total and without gender and age discrimination, 19.8% of the students reported “wheeze ever” and 28.1% reported “wheeze in the past 12 months (current wheeze).” Birth weight distribution exhibited that 8.7% of 6-7-year-old and 16.6% of 13-14-year-old age groups were under 2500 g. 30.6% of 6-7-year-old and 33% of 13-14-year-old age groups had birth weight over 3.5 kg (see Table 1).

Based on descriptive statistics analysis, the prevalence of “wheeze ever” and “wheeze in the past 12 months” in age group 6-7 years and in both genders in this group was higher than age group 13-14 years. Also, in children with birth weight under 1.5 kg (and in both genders in this group), the prevalence of “wheeze after exercise in the past 12 months” and “night cough in the past 12 months” was higher than other birth weight groups. The prevalence of studied symptoms with respect to birth weight and gender is shown in Table 2.

### 3.1. Age Group 6-7 Years

Odds ratios for “wheeze ever,” “wheeze in the past 12 months,” and “night cough in the past 12 months” in children with birth weight lower than 2.5 kg in this age group were 1.32, 1.01, and 1.32, respectively. Odds ratios for the same symptoms in children with birth weight more than 3.5 kg are 1.12, 1.11, and 1.09, respectively. Higher odds are found for males rather than females. However, odds ratio of “asthma ever” and “wheeze during or after exercise in the past 12 months” in children with birth weight lower than 2.5 kg is 0.44 and 0.67, respectively, but in group with birth weight higher than 3.5 kg, these ORs were slightly higher than that of the reference group. Similar results were obtained with respect to gender in group with birth weight higher than 3.5 kg. “Severe asthma” was the only variable with OR lower than the reference group and was significantly lower than the reference in the male group (OR = 0.12, *p* = 0.01), but the results showed a higher OR for females in the same group (OR = 1.315).

With consideration of breaking down the under 2.5 kg weight group to 3 subgroups, in subgroup males with birth weight of 1.5 to 2 kg, it was observed that the ORs of “wheeze ever” (OR = 3.90, *p* = 0.01) and “wheeze in the past 12 months” (OR = 3.90, *p* = 0.03) were significantly higher than the reference and higher than two other subgroups. This difference is also more strongly observed in the female group with birth weight lower than 1.5 kg in comparison with two other subgroups. Higher odds ratio of “night cough in the past 12 months” was observed in subgroup under 1.5 kg as compared to other groups. Similar results were detected with respect to gender (Table 3(a)).

### 3.2. Age Group 13-14 Years

ORs of all asthma symptoms and “severe asthma” were higher in group with birth weight of under 2.5 kg than the reference group. Furthermore, the ORs of “wheeze ever” (OR = 1.520, CI = 1.042–2.216, sig = 0.030) and “night cough in last 12 months” (OR = 1.774, CI = 1.205–2.613, sig = 0.004) were considerable. Females had higher odd ratios than males except in “asthma ever” symptom.

In group with birth weight higher than 3.5 kg, ORs of all symptoms were higher than the reference; however, with gender consideration, ORs of some symptoms were lower than the reference group. In female, ORs of “wheeze ever,” “asthma ever,” “wheeze during or after exercise in the past 12 months,” and “night cough” were 1.37, 2.44, 2.10, and 1.73, respectively, which were higher than ORs in male group.

With respect to subcategorization of group with birth weight under 2.5 kg, it was observed that the ORs of all symptoms except “asthma ever” were higher than the reference in all subgroups, especially with birth weight 2 to 2.5 kg. ORs of all symptoms were lower than the reference in males with birth weight of 1.5 to 2 kg. In males with birth weight under 1.5 kg, odds ratios of “wheeze during or after exercise in the past 12 months,” “asthma ever,” and “night cough in last 12 months” were higher than the reference (Table 3(b)).

*In age group 6-7 years*, “wheeze ever” ORs were lower than the reference in groups with gestational age lower than 37 weeks and more than 40 weeks; conversely, “severe asthma” ORs in both groups were 3.65 and 2.06 (higher than reference group). ORs for “wheeze in the past 12 months,” “wheeze during or after exercise in the past 12 months,” and “night cough in the past 12 months” were higher in group with gestational age more than 40 weeks with 1.11, 3.04, and 1.45, respectively. Odds ratio for “asthma ever” was lower than the reference; meanwhile, “asthma ever” OR for group with gestational age under 37 weeks was higher than reference group (Table 4).

*In age group 13-14 years*, “asthma ever” ORs, in children with gestational age less than 37 weeks and more than 40 weeks, overall and for males were higher than the reference group. However, in children with gestational age less than 37 weeks, “wheeze in the past 12 months” ORs in both groups, in general and for both genders, were lower than reference. ORs for “wheeze ever” and “night cough in the past 12 months,” generally and in males, were higher than the reference group but OR for “wheeze in the past 12 months” was lower than the reference. In the same age group with gestational age higher than 40 weeks, the “wheeze ever” OR was significantly higher than the reference group (OR = 1.81, *p* = 0.048). Also, ORs for “asthma ever” and “night cough in the past 12 months” were higher than the reference. Generally, ORs for all symptoms were higher in males than females. For women, ORs of “asthma ever,” “wheeze during or after exercise in the past 12 months,” and “night cough in the past 12 months” were lower than the reference (Table 4).

Generally, in both age groups with birth weight lower than 2.5 kg, the ORs for “wheeze ever” and “night cough in the past 12 months” were higher than reference. The ORs for “severe asthma” in girls within the age groups of 13-14 years and 6-7 years were higher than reference. The ORs of “wheeze ever” and “wheeze in the past 12 months” in the group with birth weight more than 3.5 kg, in age group 13-14 years, and ORs of “severe asthma” in boys of 6-7 years were lower than reference. Children with birth weight under 1.5 kg had higher ORs for “night cough in the past 12 months” and in the birth weight group of 1.2–2 kg ORs of “wheeze ever” and “wheeze in the past 12 months” were higher. The ORs of all symptoms were reported higher in age group of 13-14 years with birth weight between 2 and 2.5 kg, but the ORs of “severe asthma” are lower in both sexes.

With respect to gestational age, in children born before 37 weeks, in age group 6-7 years, there were higher ORs for “severe asthma” and “asthma ever” and, in age group 13-14 years, ORs for “wheeze ever,” “asthma ever,” “night cough in the past 12 months,” and “severe asthma” were higher than the reference group.

In children with gestational age more than 40 weeks, ORs of “wheeze during or after exercise in the past 12 months,” “night cough in the past 12 months,” “wheeze in the past 12 months,” and “severe asthma” in age group 6-7 years and “wheeze ever,” “asthma ever,” and “night cough in the past 12 months” in age group 13-14 years were higher than the reference group.

There were also lower ORs for “wheeze ever” and “asthma ever” for age group 6-7 years and “wheeze in the past 12 months” for age group 13-14 years who were born before 37 weeks.

## 4. Discussion

According to a meta-analysis of Auckland University from the results of ISAAC studies in 69 centers from 38 countries on more than 1000 population with more than 60% response rate, despite all the differences between centers, children with birth weight lower than 2.5 kg carried a higher risk of developing asthma symptoms in age group 6-7-years. In this study, adjusted OR for “severe asthma” was greater than “wheeze in the past 12 months”, and no significant variation was seen between sexes (interaction *p* value > 0.05) [30]. In our study on age group 6-7 years with birth weight under 2.5 kg, the ORs of “wheeze ever” and “night cough in the past 12 months” were higher and also in males when compared to the control group; also female OR of “severe asthma” was higher than the control group. In a previously mentioned study, the prevalence of “wheeze in the past 12 months” in the group with birth weight under 2.5 kg, in studied centers, ranged from 2 to 18%. In our study, the prevalence of “wheeze in the past 12 months” was evaluated in different weight subgroups and results are as follows: 21.4% in group with birth weight under 1.5 kg, 23.3% in 1.5–2 kg, and 16.5% in 2–2.5 kg. Our study also showed that low birth weight in age group 13-14 years is associated with an increase in OR for all asthma symptoms including severe asthma and ORs of “wheeze ever” (*p* = 0.030) and “night cough in the past 12 months” (*p* = 0.004) were significantly higher than reference and, like low birth weight (under 2.5 kg), it is accompanied with a higher rate of asthma and its severity in age group 13-14 years. Previous studies did not examine the role of low birth weight in age group 13-14 years.

In a cross-sectional study, based on the diagnostic criteria of ISAAC for evaluation of allergic disease symptoms in 3-year-old children with history of preterm birth or low birth weight in Japan, no significant association was found between low birth weight (under 2.5 kg) and the prevalence of wheezing and asthma. In the aforementioned study, weight group under 2.5 kg was not divided into smaller subgroups [31]. In our study, the ORs of “wheeze ever” and “night cough in the past 12 months” in 6-7-year age group with birth weight of under 1.5 kg and 1.5–2 kg were higher than the reference (2.5–3.5 kg).

In a cohort study in Denmark, Sweden, and Finland, the risk of hospitalization with severe asthma attack was considerably higher in children with low birth weight but this relationship was not seen in children with a history of preterm birth. This study did not evaluate the severity of asthma attacks, but the results of our study showed an association between severe asthma (based on asthma control) and birth weight less than 2.5 kg, in 13-14-year age group and girls of 6-7-year age group [32].

In a retrospective cohort study, the incidence of asthma was compared in children with birth weight above 2500 g and with birth weight below 2500 g. 6.7% of children with birth weight under 2500 g and 5.4% of children with birth weight more than 2500 g showed symptoms of asthma (*p* = 0.42). After propensity score matching and considerable decrease of covariate imbalance and observing the same risk in both groups, it was concluded that there is no association between the incidence of asthma and birth weight [33]. In the present study, the prevalence of “wheeze ever” in 6-7-year and 13-14-year age group is in birth weight under 1500 g, 28.6% and 10.3% are in birth weight 1500–2000 g, 32.6% and 10.9% are in group with birth weight 2500–3500 g, and 21.2% and 8.4% lastly are in the group with birth weight above 3500 g 23.6% and 9.1%, respectively.

Significantly lower ORs for “severe asthma” have been shown in the 6-7-year group with birth weight above 3.5 kg (sig = 0.044). In a cohort study which evaluated the relationship between high birth weight and risk of asthma before 6 years of age in Toronto, the risk of asthma has been shown to be reduced with high birth weight (adjusted RR 0.90, 95% Cl 0.86 to 0.93); nevertheless, the risk of asthma is slightly increased with substantial high birth weight (more than 6 kg) [34].

On the other hand, a cohort study in Finland evaluated the relationship between high birth weight with chances of atopic symptoms including asthma, until 16 years of age (based on the physician's diagnosis); results demonstrated a higher risk of asthma in children with birth weight above 4510 g and suggested that increased risk of asthma is caused by escalation in risk of atopic disorders in obese children [35].

A meta-analysis, which studied articles from 1990 to 2013, reported no increase in risk of asthma in children with birth weight more than 4000 g [36]. Furthermore, another meta-analysis, which studied 90,000 children and adults, found no association between risk of asthma and birth weight above 4 kg [29].

ISAAC study from Auckland University showed that high birth weight does not increase the risk of asthma symptoms and suggested that other studies which showed increase in ORs of asthma are caused by bias. This study did not find an increase in asthma symptoms in 6-7-year-old children with high birth weight; however, an increased risk of “night cough in the past 12 months” and “wheeze in the past 12 months” (current wheeze) was found in 13-14-year-old children with high birth weight. This discrepancy could be the result of the difference in weight grouping in our study (>3.5 kg) versus ISAAC (>4 kg). It could also be a result of the different studied age groups by ISAAC (6-7 years old) and our study (6-7 and 13-14 years old). In other words, high birth weight could cause higher risk of asthma symptoms in adolescence, but not in childhood. A decreased risk of “asthma ever” and exercise asthma in girls of 13-14 years with gestational age of more than 40 weeks has been found.

A meta-analysis for 147,252 children of 31 birth cohort studies to determine the associations of birth and infant growth characteristics with the risks of preschool wheezing (1–4 years) and school-age asthma (5–10 years) showed that lower gestational age at birth was associated with higher risks of preschool wheezing and school-age asthma (*p* < 0.05) and the associations of lower birth weight with childhood asthma were largely explained by gestational age at birth [37]. An increased risk of “severe asthma” was also found in the age group of 6-7 years with prematurity; furthermore, the study showed an increase in risk of “asthma ever,” “wheeze ever,” and “night cough” in the age group 13-14 years with gestational age earlier than 37 weeks.

This study has also provided evidence about increased risk of “severe asthma” and “wheezing during or after exercise in the past 12 months” in the age group of 6-7 years with gestational age of more than 40 weeks and also an increased risk of “asthma ever” and “wheeze ever” in the age group of 13-14 years.

## 5. Conclusion

This study showed that there is no linear association between birth weight or gestational age with severity of asthma, but the risk of “wheeze ever” is increased in both age groups with LBW and with prematurity in 13-14-year age group. There was also an increased risk of “severe asthma” in 6-7-year-old children with a history of prematurity.

## Figures and Tables

**Table 1:** Distribution of birth weight in age groups.

Birth weight	6-7 yr		13-14 yr	
	Number	%	Number	%
<1.5 kg	14	0.5	29	1.3
1.5–2 kg	43	1.4	55	2.4
2–2.5 kg	212	6.8	298	12.9
2.5–3.5 kg	1165	37.6	1104	47.9
>3.5 kg	950	30.6	760	33
Not defined	21	0.9	58	2.5
Missing	697	22.5	1250	35.2
Total	2405	100	2304	100

**Table 2:** Prevalence of asthma symptoms in different subgroups of birth weight (%).

Symptoms	Gender	BW < 1.5 kg		BW = 1.5–2 kg		BW = 2–2.5 kg		BW = 2.5–3.5 kg		BW > 3.5 kg
		6-7 yr	13-14 yr	6-7 yr.	13-14 yr	6-7 yr	13-14 yr	6-7 yr	13-14 yr	6-7 yr
Wheeze ever	Female	30	0	21.4	14.3	22.1	14.4	21.2	7.9	22.6
	Male	25	15.8	53	7.4	27.8	12.3	21.2	8.8	24.5
	Total	28.6	10.3	32.6	10.9	24.5	13.4	21.2	8.4	23.6

Wheeze in the past 12 months	Female	20	0	10.7	10.7	14.6	7.9	14.3	13.4	15.1
	Male	25	10.5	46.7	3.7	18.9	8.4	15.2	4.7	17.6
	Total	21.4	6.9	23.3	7.3	16.5	8.4	14.8	4.3	16.4

Asthma ever	Female	10	10	0	3.6	1.6	1.4	4.5	2.3	3.9
	Male		10.5	0		3.3	7.7	5.4	3.6	6.7
	Total	7.1	10.3	0	1.8	2.4		4.9	3.1	5.4

Wheeze during or after exercise in the past 12 months	Female	10	0	0	10.7	3.3	6.5	6.1	3.3	6.6
	Male	25	10.5	0	0	6.7	8.6	7.1	5.4	8.5
	Total	14.3	6.9	0	5.5	4.7	6.4	6.5	4.3	7.6

Night cough in the past 12 months	Female	20	10	7.1	17.9	16.4	17.3	15.1	7.4	18.9
	Male	50	15.8	33.3	3.7	30	8.4	20.2	7.4	18.9
	Total	28.6	13.8	16.3	10.9	22.2	12.4	17.4	7.3	18.9

**(a):** 

Symptoms	Gender	Birth weight (kg)					
		<1.5	1.5–2	2–2.5	<2.5	2.5–3.5	>3.5
Wheeze ever	Total	1.39 (0.43–4.49)	1.67 (0.87–3.22)	1.24 (0.88–1.76)	1.32 (0.97–1.80)	1.00	1.12 (0.91–1.38)
	Male	1.14 (0.12–11.03)	**3.90 (1.38–10.98)**	1.42 (0.85–2.37)	1.66 (1.04–2.63)	1.00	1.17 (0.875–1.58)
	Female	1.48 (0.38–5.79)	0.94 (0.37–2.37)	1.11 (0.690–1.780)	1.10 (0.72–1.67)	1.00	1.07 (0.80–1.44)

Wheeze in the past 12 months	Total	0.95 (0.24–3.70)	1.38 (0.61–3.10)	0.94 (0.67–1.45)	1.01 (0.68–1.48)	1.00	1.11 (0.85–1.45)
	Male	0.74 (0.08–7.25)	**3.90 (1.10–13.70)**	1.15 (0.60–2.18)	1.39 (0.79–2.45)	1.00	1.17 (0.81–1.70)
	Female	1.08 (0.19–6.01)	0.54 (0.15–1.96)	0.795 (0.44–1.44)	0.76 (0.45–1.31)	1.00	1.065 (0.725–1.565)

Severe asthma	Total	0.20 (0.02–1.84)	*∗*	0.75 (0.20–2.835)	0.73 (0.22–2.42)	1.00	0.60 (0.25–1.43)
	Male	0.02 (0.01–0.37)	*∗*	0.09 (0.01–1.04)	0.08 (0.01–0.08)	1.00	0.12 (0.01–0.94)
	Female	*∗*	*∗*	**2.47 (0.300–20.25)**	**3.12 (0.38–25.54)**	1.00	1.315 (0.42–4.125)

Asthma ever	Total	1.47 (0.19–11.46)	*∗*	0.46 (0.18–1.16)	0.44 (0.19–1.04)	1.00	1.07 (0.725–1.58)
	Male	*∗*	*∗*	0.57 (0.17–1.90)	0.47 (1.14–1.57)	1.00	1.280 (0.67–2.15)
	Female	2.18 (0.27–17.82)	*∗*	0.36 (0.85–1.53)	0.42 (0.13–1.40)	1.00	0.84 (0.45–1.545)

Wheeze during or after exercise in the past 12 months	Total	2.41 (0.53–10.99)	*∗*	0.70 (0.36–1.38)	0.67 (0.36–1.25)	1.00	1.162 (0.83–1.63)
	Male	4.21 (0.43–41.46)	*∗*	0.91 (0.37–2.230)	0.88 (0.38–2.04)	1.00	1.245 (0.79–1.97)
	Female	1.66 (0.20–13.40)	*∗*	0.52 (0.18–1.49)	0.497 (0.19–1.28)	1.00	1.08 (0.66–1.78)

Night cough in the past 12 months	Total	1.92 (0.50–6.21)	1.00 (0.435–2.29)	1.35 (0.94–1.94)	1.32 (0.950–1.84)	1.00	1.09 (0.87–1.37)
	Male	**3.80 (0.53–27.30)**	**2.11 (0.69–6.43)**	**1.655 (1.00–2.730)**	1.770 (1.12–2.80)	1.00	0.94 (0.69–1.285)
	Female	1.35 (0.28–6.46)	0.450 (0.105–1.94)	1.10 (0.65–1.87)	1.00 (0.61–1.62)	1.00	1.30 (0.94–1.80)

^*∗*^Shows models that could not converge.

Bold font shows significant OR.

**(b):** 

Symptoms	Gender	Birth weight (kg)					
		<1.5	1.5–2	2–2.5	<2.5	2.5–3.5	>3.5
Wheeze ever	Total	1.00 (0.30–3.39)	1.23 (0.51–2.99)	**1.645 (1.09–2.47)**	1.52 (1.04–2.22)	1.00	1.09 (0.78–1.52)
	Male	1.60 (0.45–5.75)	0.83 (0.19–3.67)	1.59 (0.895–2.83)	1.48 (0.87–2.50)	1.00	0.89 (0.57–1.41)
	Female	*∗*	1.66 (0.54–5.07)	1.72 (0.97–3.075)	1.58 (0.92–2.72)	1.00	1.37 (0.84–2.23)

Wheeze in the past 12 months	Total	1.17 (0.24–5.61)	1.265 (0.41–3.92)	1.62 (0.93–2.81)	1.52 (0.91–2.54)	1.00	1.05 (0.65–1.695)
	Male	1.91 (0.35–10.37)	0.597 (0.07–4.975)	1.44 (0.685–3.05)	1.365 (0.68–2.73)	1.00	1.21 (0.65–2.24)
	Female	*∗*	1.935 (0.49–7.64)	1.87 (0.82–4.24)	1.74 (0.82–3.695)	1.00	0.85 (0.40–1.83)

Severe asthma	Total	0.47 (0.05–4.19)	*∗*	2.275 (0.49–10.57)	1.96 (0.48–7.21)	1.00	1.43 (0.48–4.235)
	Male	*∗*	*∗*	*∗*	*∗*	1.00	1.96 (0.37–10.42)
	Female	0.15 (0.01–1.855)	*∗*	1.21 (0.23–6.29)	1.00 (0.24–4.20)	1.00	1.15 (0.27–4.80)

Asthma ever	Total	2.98 (0.85–10.44)	0.58 (0.08–4.37)	1.66 (0.87–3.16)	1.61 (0.89–2.91)	1.00	1.42 (0.86–2.34)
	Male	2.71 (0.58–12.695)	*∗*	**2.555 (1.21–5.39)**	2.175 (1.07–4.41)	1.00	0.94 (0.48–1.86)
	Female	3.47 (0.41–29.64)	1.42 (0.18–11.425)	0.56 (0.12–2.53)	0.87 (0.28–2.755)	1.00	**2.44 (1.14–5.20)**

Wheeze during or after exercise in the past 12 months	Total	1.32 (0.30–5.76)	1.30 (0.39–4.37)	1.46 (0.83–2.56)	1.42 (0.85–2.385)	1.00	1.27 (0.825–1.955)
	Male	1.78 (0.39–8.12)	*∗*	1.25 (0.58–2.71)	1.14 (0.555–2.32)	1.00	0.865 (0.48–1.55)
	Female	*∗*	3.29 (0.90–12.10)	1.85 (0.80–4.26)	1.93 (0.90–4.14)	1.00	**2.10 (1.09–4.06)**

Night cough in last 12 months	Total	1.69 (0.57–5.01)	1.55 (0.63–3.795)	**1.83 (1.20–2.78)**	1.77 (1.205–2.61)	1.00	**1.46 (1.045–2.03)**
	Male	1.99 (0.55–7.195)	0.55 (0.07–4.20)	1.30 (0.67–2.51)	1.27 (0.70–2.31)	1.00	1.23 (0.78–1.95)
	Female	1.08 (0.13–8.75)	2.555 (0.90–7.23)	**2.40 (1.375–4.20)**	2.33 (1.39–3.92)	1.00	**1.73 (1.07–2.80**)

^*∗*^Show models that could not converge. Bold font shows significant OR.

**Table 4:** Odds ratios (95% confidence interval) for gestational age categories (reference category 37–40 wk) for symptoms of asthma, 6-7 yr and 13-14 yr.

Symptoms	Gender	6-7 years			13-14 years		
		Gestational age (CI) week					
		<37 weeks	37–40 weeks	>40 weeks	<37 weeks	37–40 weeks	>40 weeks
Wheeze ever	Total	0.98 (0.69–1.37)	1.00	0.89 (0.56–1.42)	1.22 (0.75–1.98)	1.00	**1.81 (1.01–3.25)**
	Male	0.88 (0.54–1.44)	1.00	0.69 (0.345–1.39)	1.560 (0.82–2.97)	1.00	**2.19 (1.00–4.78)**
	Female	1.08 (0.67–1.74)	1.00	1.12 (0.60–2.07)	0.90 (0.43–1.91)	1.00	1.455 (0.60–3.55)

Wheeze in the past 12 months	Total	0.82 (0.52–1.29)	1.00	1.11 (0.61–2.03)	0.74 (0.37–1.48)	1.00	0.60 (0.22–1.650)
	Male	0.76 (0.41–1.41)	1.00	1.17 (0.49–2.81)	0.47 (0.3021.83)	1.00	0.56 (0.15–2.06)
	Female	0.91 (0.47–1.75)	1.00	1.06 (0.46–2.45)	0.74 (0.25–2.17	1.00	0.68 (0.14–3.300)

Severe asthma	Total	3.65 (0.47–28.26)	1.00	2.06 (0.26–16.205)	1.70 (0.35–8.34)	1.00	*∗*
	Male	*∗*	1.00	0.60 (0.08–5.46)	*∗*	1.00	*∗*
	Female	2.325 (0.28–19.05)	1.00	*∗*	0.10 (0.17–5.255)	1.00	*∗*

Asthma ever	Total	1.13 (0.59–2.16)	1.00	0.640 (0.23–1.805)	1.28 (0.60–2.715)	1.00	1.33 (0.49–3.57)
	Male	1.14 (0.48–2.67)	1.00	0.59 (0.14–2.53)	1.615 (0.63–4.16)	1.00	1.885 (0.590–6.02)
	Female	1.12 (0.42–3.02)	1.00	0.71 (0.16–3.07)	0.880 (0.24–3.205)	1.00	0.63 (0.80–5.01)

Wheeze during or after exercise in the past 12 months	Total	0.80 (0.42–1.55)	1.00	**3.04 (1.76–5.24)**	0.92 (0.47–1.78)	1.00	0.95 (0.39–2.30)
	Male	0.91 (0.39–2.11)	1.00	**3.24 (1.54–6.845)**	0.96 (0.40–2.32)	1.00	1.13 (0.37–3.42)
	Female	0.675 (0.23–1.96)	1.00	2.82 (1.27–6.260)	0.870 (0.32–2.40)	1.00	0.75 (0.16–3.23)

Night cough in the past 12 months	Total	0.97 (0.66–1.41)	1.00	1.45 (0.930–2.255)	1.20 (0.73–1.96)	1.00	1.18 (0.61–2.27)
	Male	1.26 (0.77–2.065)	1.00	1.340 (0.70–2.555)	1.27 (0.64–2.530)	1.00	1.63 (0.70–3.77)
	Female	0.69 (0.375–1.25)	1.00	1.55 (0.845–2.85)	1.145 (0.57–2.31)	1.00	0.75 (0.25–2.240)

^*∗*^Shows models that could not converge

Bold font shows significant OR.
